# Altered systemic levels of acute phase proteins in tuberculous lymphadenitis and modulation after treatment

**DOI:** 10.1371/journal.pone.0233426

**Published:** 2020-05-29

**Authors:** Gokul Raj Kathamuthu, Kadar Moideen, Nathella Pavan Kumar, Rathinam Sridhar, Dhanaraj Baskaran, Subash Babu

**Affiliations:** 1 National Institutes of Health-NIRT-International Center for Excellence in Research, Chennai, India; 2 National Institute for Research in Tuberculosis (NIRT), Chennai, India; 3 Government Stanley Medical Hospital, Chennai, India; 4 Laboratory of Parasitic Diseases, National Institute of Allergy and Infectious Diseases, National Institutes of Health, Bethesda, Maryland, United States of America; Karolinska Institutet, SWEDEN

## Abstract

**Background:**

Pulmonary tuberculosis (PTB) is characterized by elevated levels of acute phase proteins (APPs), but their association with tuberculous lymphadenitis (TBL) is poorly studied.

**Methods:**

We examined the systemic levels of APPs (alpha-2-macroglobulin [⍺-2MG], serum amyloid A [SAA], C-reactive protein [CRP] and haptoglobin [Hp]) in TBL, PTB, latent tuberculosis (LTB) and healthy controls (HC) at baseline and in TBL after the completion of anti-tuberculosis treatment (ATT). We have also examined the association of these proteins with lymph node (LN) size, culture grade and multiple versus single LN involvement.

**Results:**

TBL individuals exhibited increased systemic levels of ⍺-2MG, SAA, CRP and Hp in comparison to HCs and increased CRP levels in comparison to LTB individuals. TBL individuals also exhibited decreased systemic levels of Hp compared to PTB individuals. APPs were not significantly associated with LN size, LN involvement and culture grade, indicating a lack of association with disease severity. Following ATT, post-treatment levels of ⍺-2MG, CRP and Hp were significantly diminished compared to pre-treatment levels.

**Conclusion:**

TBL disease is characterized by altered levels of APPs at baseline and modulated following treatment, indicating the presence of systemic inflammation.

## Introduction

Globally, tuberculosis (TB) still remains a major health problem causing high morbidity and mortality [1]. Tuberculous lymphadenitis (TBL) is a common manifestation of extrapulmonary tuberculosis (EPTB) with increased prevalence in developing countries [2, 3]. However, the mechanism of dissemination is still not clear and studies reveal that mycobacteria may mostly spread through the hematogenous route [4]. TBL diagnosis is difficult and often associated with other pathologic processes which yield inconsistent physical and laboratory findings. The diagnosis can be made either via radiologic examination or fine-needle aspiration cytology (FNAC)/ histological examination of dissected cervical lymph nodes. A broad array of specific and non-specific immunological responses is assumed to contribute to the differential outcomes in TBL disease [5, 6]. TBL has been associated with altered levels of certain pro-inflammatory, type 1 and type 17 cytokines in the serum or plasma [7, 8]. However, in response to TB disease/infection, the host defense also triggers systemic inflammation as well as influences certain physiological, systemic and metabolic alterations. These profound changes are known as acute phase responses (APR) and often lead to abnormal production of various plasma proteins released into the bloodstream [9, 10].

Of note, some of these inflammatory biomarkers (C reactive protein (CRP), neopterin, beta 2 macroglobulin) have been used for the evaluation of therapeutic monitoring, for the detection of disseminated mycobacterial disease, persistent culture positivity, radiological resolution, and specifically for the delineation of tuberculosis from malignancy [11]. It is also noteworthy to understand that due to the difficulties in EPTB diagnosis, initiation of different ATT treatment occurs without reliable TB diagnosis [12]. Hence, discovery of reliable biomarkers without using tissue or sputum sample and with high diagnostic value would be really important in the context of EPTB diagnosis.

The major APPs are alpha 2 macroglobulin (⍺-2MG), serum amyloid A (SAA), C-reactive protein (CRP) and haptoglobin (Hp) and their synthesis is tightly regulated by inflammatory cytokines like interleukin (IL)-1 and IL-6 [13]. APPs have an imperative role in scavenging extracellular hemoglobin, free radicals, iron, and are majorly considered as components of innate immunity with antibacterial and antiviral potential [14, 15]. ⍺-2MG is a component of innate immune system and mediates potential role in tissue remodeling and immune system regulation [16]. ⍺-2MG also facilitates the innate immune defence mechanism against pathogens in plasma and vertebrate tissues [17]. SAA also displays a wide variety of immune functions by inducing the synthesis of numerous cytokines and chemokines as well as activating inflammatory cascades [18]. SAA levels are also enhanced in neoplasia, injuries, trauma, infection and acute phase of inflammation [19]. C-reactive protein (CRP), an established biomarker of systemic inflammation, has been described to reflect TB disease severity [20]. CRP has a vital role as an indicator of immune system activity during inflammatory responses [21, 22]. Hp plays a crucial role in immune regulatory and inflammatory conditions by modulating the prostaglandin synthesis [23]. Hp levels are known to become altered because it is the main protein found in human plasma for hemoglobin (Hb) binding, which reduces the harmful physiologic and biochemical outcome of extracellular Hb [24]. Previous studies have shown the association of APPs with respect to pulmonary TB and pneumonia infection [15, 25–29]. Despite this, their collective role or their systemic levels have not been examined in TBL disease/infection. In order to determine whether systemic inflammation is associated with TBL, we examined the levels of these APPs at baseline and upon after the completion of ATT.

## Methods

### Study design

This study was conducted at NIRT, Chennai, India and the patients were recruited from hospitals and community screening in and around Chennai. The study was approved by Institutional Review Board (NIRTIEC2010007) of National Institute for Research in Tuberculosis (NIRT) Chennai, Tamil Nadu, India. The written informed consent was obtained from all the study participants before enrolment. The plasma samples from TBL (n = 44), PTB (n = 44), latent TB (LTB, n = 44) and HC (n = 44) individuals were collected and used in the study. TBL individuals were diagnosed either by histopathology positivity or bacteriology examination consisting of GeneXpert or culture positivity for *Mycobacterium tuberculosis* (Mtb). Pulmonary TB individuals were selected based on the positive diagnosis for smear and culture examination for Mtb. All TBL individuals had only cervical lymphadenopathy. LTB individuals were diagnosed on the basis of positivity for tuberculin skin test (TST) and QuantiFERON TB-Gold in tube ELISA and absence of chest radiograph abnormalities and pulmonary symptoms. TST positive result was defined as an induration at the site of tuberculin inoculation of at least 10mm in diameter to minimize the false positivity due to environmental mycobacteria exposure. Only those were positive for both TST and QFT was considered as LTB and negative for both were considered as healthy controls (HC). Both LTB and HC samples were collected from the screening of community people. The status of lymphadenopathy in both PTB and LTB individuals was assessed by physical examination. Those PTB or LTB individuals with enlargement of the lymph nodes have been removed from the study as per exclusion criteria. Blood samples from PTB, LTB and HC individuals were collected only at baseline. All the individuals were HIV negative and not under any steroid treatment during the study period. All individuals were also not afflicted with any other chronic viral or bacterial infection based on medical history and physical examination. The duration of anti-tuberculosis treatment (ATT) was 6 months and blood samples from TBL individuals were collected again after the completion of ATT. All the TBL individuals were cured from the disease which was confirmed by the disappearance of lymph node expansion analysed on X-ray and CT-scans.

### Measurement of APPs

The plasma levels of acute phase proteins (⍺-2MG, SAA, CRP, Hp) were measured using ELISA kits, according to the manufacturer’s instructions. All the ELISA kits were purchased from R&D Systems (DuoSet) except Hp which was purchased from My BioSource. The threshold detection limit for different proteins was as follows: ⍺-2MG- 0.625 ng/ml, SAA- 1.563 ng/ml, CRP- 15.625 pg/ml and Hp- 3.125 ng/ml.

### Data analysis

The Kruskal-Wallis non-parametric test with Dunn’s multiple comparisons was used to measure the statistical differences between TBL, PTB, LTB and HC individuals. Median, first (25% Percentile) and third (75% Percentile) quartiles were calculated using descriptive (column) statistics. Mann-Whitney U test was used to analyse the significant difference in lymph node (LN) culture grade (CG), LN size, multiple (M) versus single (S) LN involvement. Linear trend analysis (one-way ANOVA) was used to assess the association with bacterial burdens. The pre-and post-treatment levels were compared using the Wilcoxon signed rank test. The statistical analysis was performed using GraphPad Prism 8.0 (GraphPad Software Inc., San Diego, CA).

## Results

### Study demographics

The demographic details of the study individuals are listed in Table 1. There were no significant differences in the age and culture/smear status (except gender) between the study groups.

**Table 1:** Demographics of the study individuals.

Study Demographics	TBL	PTB	LTB	HC	P value
Number of subjects recruited (n)	44	44	44	44	
Gender (M/F)	18/26*	29/15*	15/29*	20/24*	p = 0.01
Median age in years (Range)	30 (18–51)	31 (19–54)	32 (21–62)	34 (21–55)	
Culture/ smear grade (0/1+ /2+ /3+)	8/34/2/0*	0/10/15/19*	Not done	Not done	NS
QuantiFERON-TB Gold	Not done	Not done	Positive	Negative	

*Calculated using chi-square tests; NS = non-significant.

### Altered systemic levels of APPs in TBL

**Fig 1:**
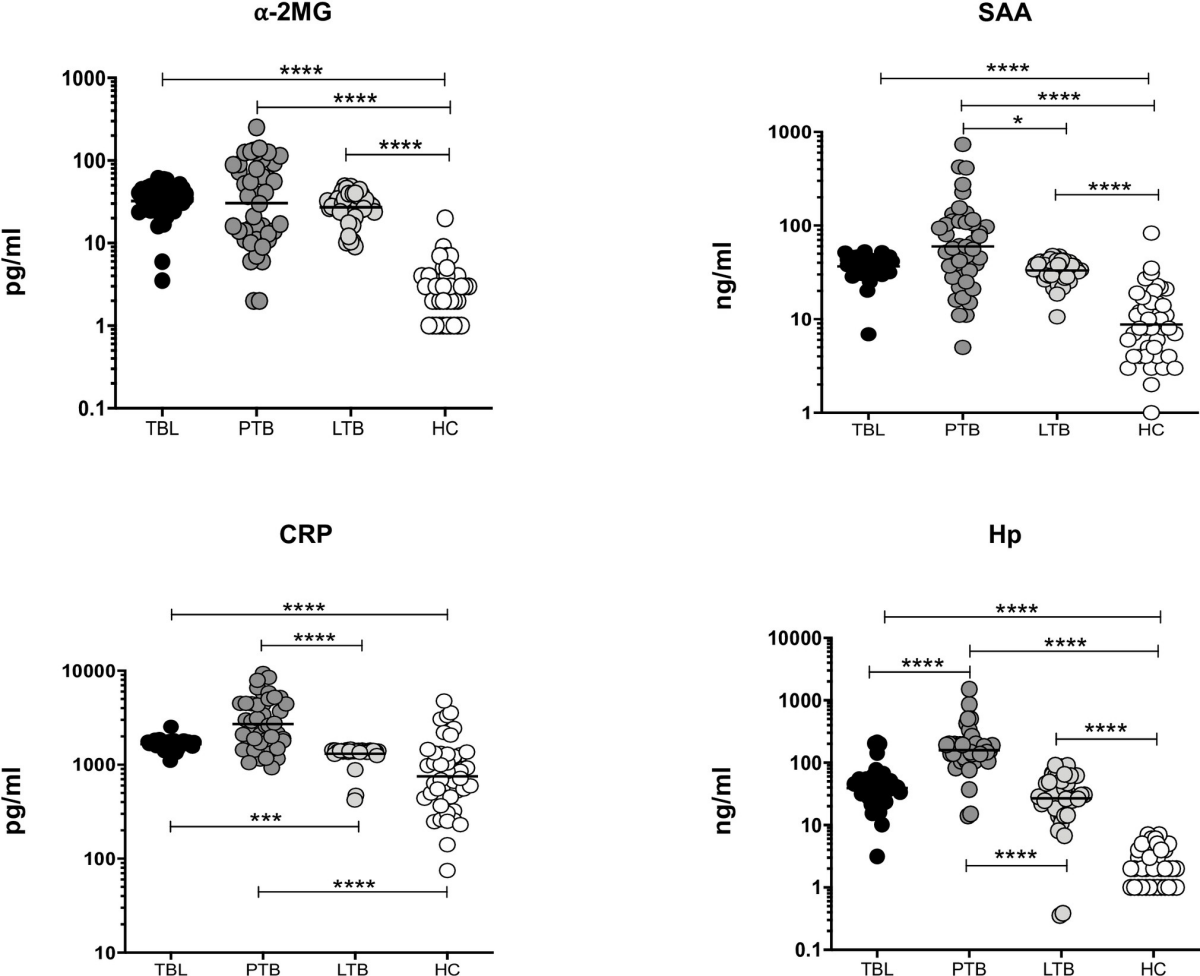
TBL is characterised by altered plasma levels of acute phase proteins. The plasma levels of acute phase proteins (⍺-2MG, SAA, CRP and Hp) were measured in TBL (n = 44), PTB (n = 44), LTB (n = 44) and HC (n = 44) individuals. We have shown our data as scatter plots with each circle representing a single individual and medians depicted with a bar. P values (*p < 0.05, ***p < 0.001, ****p < 0.0001) were measured using the Kruskal-Wallis test with Dunn’s multiple comparisons.

We examined the plasma levels of APPs (⍺-2MG, SAA, CRP, and Hp) in TBL, PTB, LTB and HC individuals (Fig 1). We show that TBL, PTB and LTB individuals are associated with enhanced plasma levels of ⍺-2MG (median of TBL is 35.08 pg/ml, 37.50 pg/ml in PTB and 30.12 pg/ml in LTB compared to 3.0 pg/ml in HC), and SAA (median of 38.87 ng/ml in TBL, 58.50 ng/ml in PTB, 35.17 ng/ml in LTB compared to 8.5 ng/ml in HC) compared to HC individuals. SAA plasma levels were significantly higher in PTB (58.50 ng/ml) than LTB (35.17 ng/ml) individuals. In addition, the systemic levels of CRP were significantly increased in TBL and PTB individuals when compared to LTB and HC (median of 1692 pg/ml in TBL, 2620 pg/ml in PTB compared to 1393 pg/ml in LTB and 781.5 pg/ml in HC) individuals. Finally, in TBL the circulating levels of Hp were significantly diminished compared to PTB and increased compared to HC (median of 42.51 ng/ml in TBL, compared 166.0 ng/ml in PTB and 2.0 ng/ml in HC) individuals. The systemic levels of Hp were also significantly increased in PTB and LTB (median of 166.0 ng/ml in PTB and 30.38 ng/ml in LTB compared to 2.0 ng/ml in HC) individuals compared to HC individuals. The first (25% Percentile) and third (75% Percentile) quartile range are given in Table 2. The fold change in the levels of APPs in TBL, PTB and LTB compared to HCs is shown in Table 3.

**Table 2:** The first (25% Percentile) and third (75% Percentile) quartile of the study population.

APP	TBL		PTB		LTB		HC	
	25% Percentile	75% Percentile	25% Percentile	75% Percentile	25% Percentile	75% Percentile	25% Percentile	75% Percentile
SAA	31.81	44.53	32.25	110.3	28.75	40.53	4.250	46.50
A2MG	26.89	44.91	13.25	89.75	23.92	36.73	1.0	4.0
CRP	1561.0	1763.0	1701.0	4488.0	1362.0	1417.0	441.8	1316.0
HP	24.76	54.43	123.0	198.0	21.41	61.64	1.0	5.0

**Table 3:** The fold change of APPs in TBL, PTB and LTB individuals compared to HC individuals.

APP	TBL	PTB	LTB
SAA	4.1680	14.361	2.309
A2MG	12.769	12.060	10.728
CRP	2.198	3.601	1.733
HP	17.428	70.677	11.987

### APPs are not significantly associated with TBL culture grade, lymph node size, multiple versus single lymph node and bacterial burden

**Fig 2:**
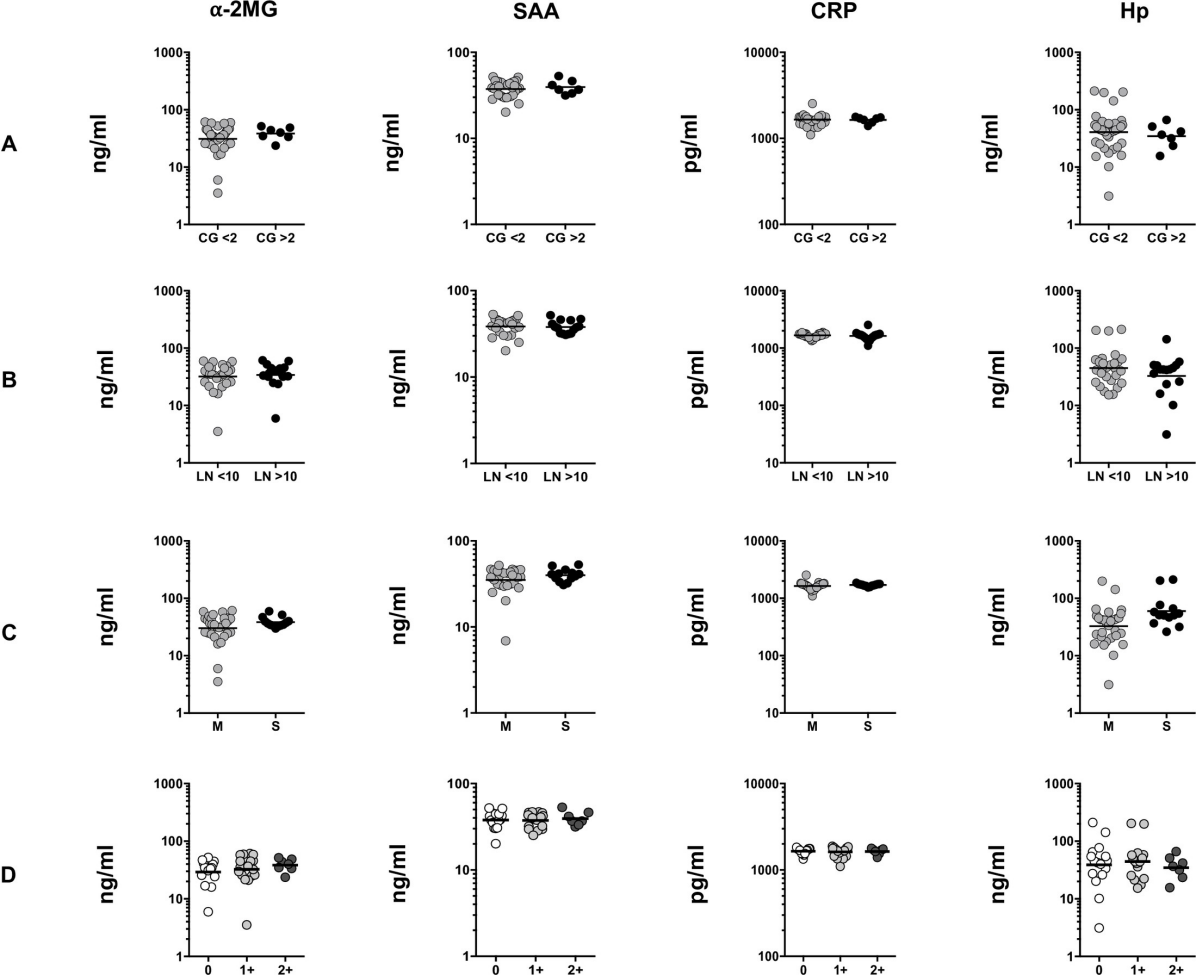
APPs are not associated with any significant relationship with lymph node (LN) culture grade, size, multiple versus single LN involvement and disease severity. (A) The plasma levels of APPs in TBL individuals with different LN culture grades (B) Levels of APPs are shown in TBL individuals with two clusters of LN (we calculated the LN size using their vertical and horizontal length and represented as < or > 10 cm) (C) The plasma levels of APPs in TBL individuals with multiple versus single LN. P values were calculated using the Mann-Whitney U test. (D) The association between systemic levels of APPs were correlated with bacillary culture grades using linear trend analysis (one-way ANOVA). The data were examined and shown as scatter plots with each circle representing a single individual.

To examine the association between the systemic levels of APPs with bacterial burdens and disease severity, we examined the plasma levels of APPs and compared them with respective culture grades, lymph node size and number among TBL individuals. As shown in Fig 2A, no significant difference was observed between the APPs in individuals with different culture grades. In addition, as shown in Fig 2B, no significant difference existed between the plasma levels of APPs in the two lymph node clusters (size of the LN was calculated using vertical and horizontal length < or > 10 cm). Similarly, none of the APPs showed any significant difference between the individuals with multiple or single lymph nodes (Fig 2C). Finally, there was no association between the APPs with the respective culture grades of TBL individuals (Fig 2D).

### Effect of ATT on APPs in TBL

**Fig 3:**
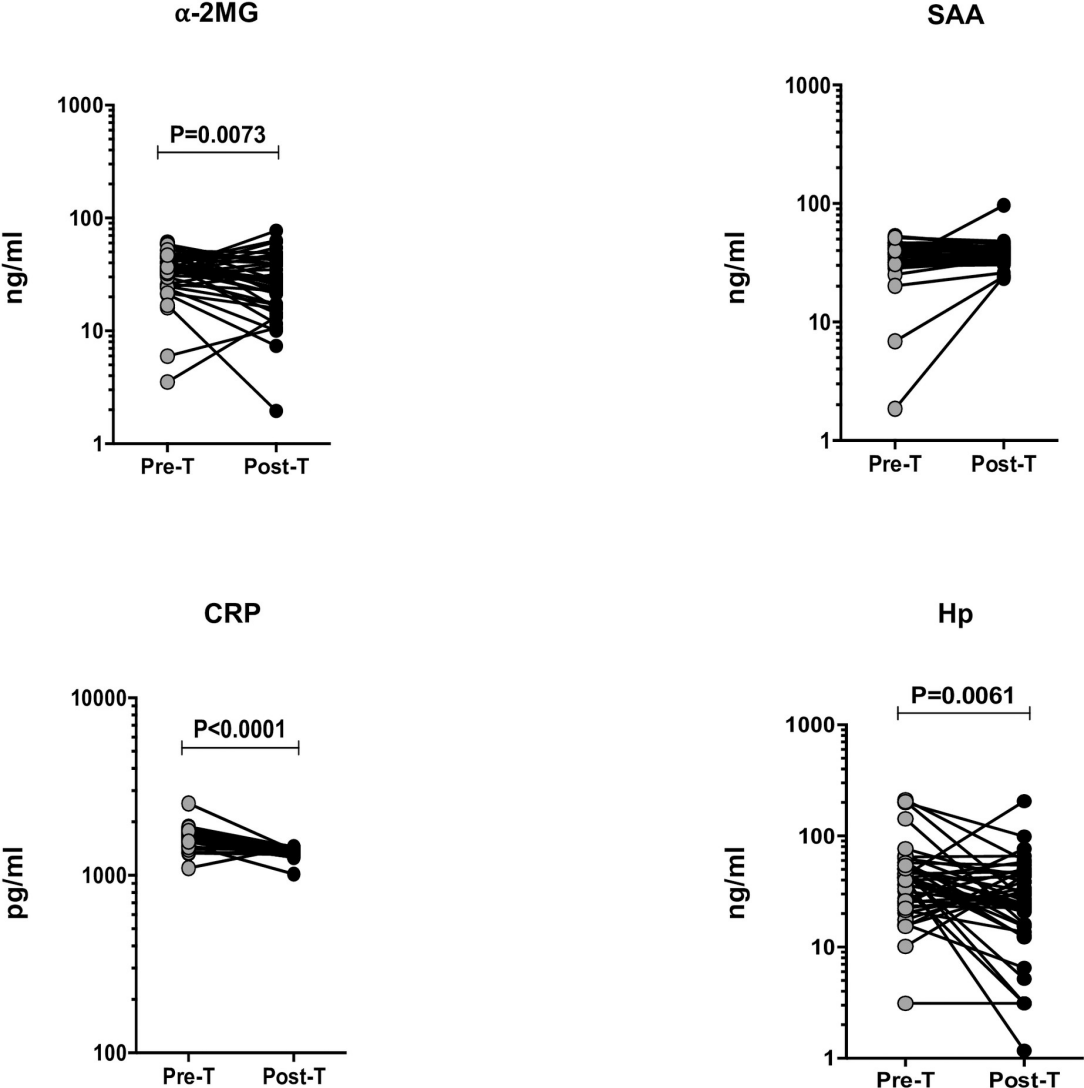
Treatment induced changes of APPs in TBL. The plasma levels of acute phase proteins (⍺-2MG, SAA, CRP and Hp) were measured in TBL individuals before (pre-T) and at the end of ATT (post-T). We have shown our data as line graphs with each line indicating a single individual. Wilcoxon signed rank test were used to measure the P values.

To determine the effect of ATT on APPs in TBL individuals, we measured the pre versus post-treatment levels of APPs (Fig 3). The post-treatment plasma levels of ⍺-2MG (median of 35.08 ng/ml in pre-T compared to 27.65 ng/ml in post-T), CRP (median of 1692 pg/ml in pre-T compared to 1398 pg/ml in post-T) and Hp (median of 42.51 pg/ml in pre-T compared to 25.31 pg/ml in post-T) were significantly diminished when compared to pre-treatment levels. In contrast, no significance was found between the pre and post-treatment plasma levels of SAA1 (median of 38.87 ng/ml in pre-T compared to 36.88 ng/ml in post-T). The first (25% Percentile) and third (75% Percentile) quartile range between TBL pre-T and post-T are given in Table 4. The fold change in APPs in post-T levels compared to pre-T levels is shown in Table 5.

**Table 4:** The first (25% Percentile) and third (75% Percentile) quartile among TBL pre-T and post-T individuals.

APP	TBL pre-T		TBL post-T	
	25% Percentile	75% Percentile	25% Percentile	75% Percentile
SAA	31.81	44.53	32.63	41.57
A2MG	26.89	44.91	17.20	39.88
CRP	1561.0	1763.0	1348.0	1430.0
HP	24.76	54.43	15.44	44.46

**Table 5:** The fold change of pre-treatment compared to post-treatment levels of APPs.

APP	TBL pre-T	TBL post-T	Fold change
SAA	36.60	32.03	1.142
A2MG	32.41	23.48	1.380
CRP	1656	1378	1.201
HP	39.11	23.29	1.679

## Discussion

APPs are generic serum proteins shown to be present at higher levels in active TB disease [30]. However, there are very few research studies that examined the systemic levels of APPs in EPTB, especially in TBL disease/infection [12, 15, 19]. Hence, this present study investigates the plasma levels of different APPs in TBL, PTB, LTB and HC individuals and their post-treatment modulation upon completion of ATT. Our findings reveal that the plasma levels of APPs were significantly altered during infection and they were modulated after the completion of ATT. Elevated plasma levels of ⍺-2MG in TBL and PTB individuals compared to HC individuals indicate hemodynamic alterations or inflammation occurring because of tissue damage at the infection site. This is reflective of a previous report in children with PTB or EPTB, where plasma levels of ⍺-2MG were heightened when compared to healthy controls [19, 31]. Moreover, after the administration of ATT, the post-treatment plasma levels of ⍺-2MG were significantly modulated when compared to pre-treatment levels. This observation signifies chronic inflammation stimulated by Mtb was decreased in TBL individuals.

Similar to ⍺-2MG, other APP (SAA) levels were also enhanced in TBL, PTB and LTB individuals compared to HCs. Higher serum levels of SAA protein were reported in PTB patients up on comparison with healthy controls [21, 32]. SAA may function as an opsonin or aid in cell recruitment to the inflammatory milieu. Further, these nonspecific inflammatory markers are chiefly produced by the liver which was demonstrated in TB and also in other diseases [33, 34]. Also, previously it has been suggested that SAA protein acts as a more profound marker of inflammation than the CRP and both were useful in elimination of infection by binding to the cell wall of microbes [35]. Herein, our observations reinforce that SAA was higher in the disease infected groups, indicating the ongoing inflammation. However, in TBL, their post-treatment levels were not modulated after the completion of ATT. This could perhaps reflect a slower kinetics in the modulation of different APPs post-treatment.

Next, we show that CRP plasma levels were also enhanced in TBL and PTB group than the LTB and HCs. Hence, the higher levels might suggest the extent of Mtb infection associated with diseased individuals. CRP has been described as a candidate biomarker for active TB disease and also in other infections as well [36, 37]. CRP also acts as an activation marker, acute phase reactant and can be effectively used as the marker for treatment monitoring. Previous studies have reported a gradual reduction in the plasma CRP levels upon ATT in PTB with improved radiographic features after 2 months of ATT [27]. Likewise, in our study the CRP levels were significantly modulated at the post-treatment time point. Future studies with point-of-care assays of CRP should provide useful information on the utility of CRP as a point of care test for TBL. Hence, we postulate CRP may serve as a marker for distinguishing TBL from LTB and HCs in monitoring the treatment progression for TBL disease.

Finally, we also examined the systemic levels of Hp between TBL, PTB, LTB and HC individuals and the levels were reduced in TBL compared to PTB individuals. Their systemic levels were higher in PTB when compared to LTB and HCs. Hp recruits the neutrophils via the activated endothelial cells and platelets for free radical quenching, tissue repair and regeneration [22]. Our results corroborate the above findings; a reduction in the post-treatment plasma levels of Hp was observed when compared to pre-treatment levels. We also examined the association of these APPs with their respective culture grade, lymph node size, multiple versus single lymph node status and bacterial burden. However, we could not find any significant association with any of these APPs with the above parameters. Some patients did exhibit increased levels of APPs following ATT, although the reason behind this needs further investigation.

In conclusion, our study is one of the first to systematically assess different APPs (⍺-2MG, SAA, CRP, and Hp) in TBL disease/infection. The altered levels of APPs could possible reflect the activation of host defence mechanism to eliminate the detrimental effects produced by TB bacteria in TBL individuals. Our study has only focused on showing the differences in the plasma levels of APPs between TBL and other groups. Overall, APPs might be involved in the immunomodulatory responses associated with active TBL disease which in turn changes after the completion of ATT.

## Supporting information

S1 Data (XLS)

## References

[1] WHO tuberculosis global tuberculosis report 2016. http://www.who.int/mediacentre/factsheets/fs104/en/.

[2] Hegde S, Rithesh KB, Baroudi K, Umar D. Tuberculous Lymphadenitis: Early Diagnosis and Intervention. J Int Oral Health. 2014; 6(6):96–98. . 25628495 PMC4295467

[3] Sandhu GK. Tuberculosis: Current Situation, Challenges and Overview of its Control Programs in India. J Glob Infect Dis. 2011; 3(2):143–150. 10.4103/0974-777X.81691 21731301 PMC3125027

[4] Volkman HE, Pozos TC, Zheng J, Davis JM, Rawls JF, et al Tuberculous granuloma induction via interaction of a bacterial secreted protein with host epithelium. Sci. 2010; 327(5964):466–469. 10.1126/science.1179663 20007864 PMC3125975

[5] Mohapatra PR, Janmeja AK. Tuberculous lymphadenitis. J Assoc Phys India. 2009; 57:585–590. . 20209720

[6] Handa U, Mundi I, Mohan S. Nodal tuberculosis revisited: a review. J Infect Dev Ctries. 2012; 6(1):6–12. 10.3855/jidc.2090 . 22240421

[7] Mustafa T, Brokstad KA, Mfinanga SG, Wiker HG. “Multiplex analysis of pro- or anti-inflammatory serum cytokines and chemokines in relation to gender and age among tanzanian tuberculous lymphadenitis patients. Tubercul Res. 2015; 2015:561490 10.1155/2015/561490 26060581 PMC4427821

[8] Kathamuthu GR, Moideen K, Bhaskaran D, Sekar G, Sridhar R, Vidyajayanthi B, et al Reduced systemic and mycobacterial antigen-stimulated concentrations of IL-1β and IL-18 in tuberculous lymphadenitis. Cytokine. 2017; 90:66–72. 10.1016/j.cyto.2016.10.013 27794266 PMC5291808

[9] Kushner I, Broder ML, Karp D. Control of acute phase response. Serum C-reactive protein kinetics after acute myocardial infarction. J Clin Invest. 1978; 61(2):235–242. 10.1172/JCI108932 621273 PMC372532

[10] Cray C, Zaias J, Altman NH. Acute phase response in animals: a review. Comp Med. 2009; 59(6):517–526. . 20034426 PMC2798837

[11] Sharma V, Mandavdhare HS, Lamoria S, Singh H, Kumar A. Serial C-reactive protein measurements in patients treated for suspected abdominal tuberculosis. Dig Liver Dis. 2018; 50(6):559–562. 10.1016/j.dld.2017.12.008 29301734

[12] Albuquerque VVS, Kumar NP, Fukutani KF, Vasconcelos B, Arriaga MB, Silveira-Mattos PS, et al Plasma levels of C-reactive protein, matrix metalloproteinase-7 and T lipopolysaccharide-binding protein distinguish active pulmonary or extrapulmonary tuberculosis from uninfected controls in children. Cyt. 2019; 123:154773 10.1016/j.cyto.2019.154773 31299414

[13] Janciauskiene S, Welte T, Mahadeva R. Acute Phase Proteins: Structure and function relationship, acute phase proteins—regulation and functions of acute phase pProteins, Prof. Francisco Veas (Ed.), ISBN: 2011: 978-953-307-252-254.

[14] Medzhitov R. Recognition of microorganisms and activation of the immune response. Nat. 2007; 449(7164):819–826. 10.1038/nature06246 17943118

[15] Immanuel C, Acharyulu GS, Kannapiran M, Segaran R, Sarma GR. Acute phase proteins in tuberculous patients. Indian J Chest Dis Allied Sci. 1990; 32(1):15–23. 1702755

[16] Rehman AA, Ahsan H, Khan FH. α-2-Macroglobulin: a physiological guardian. J Cell Physiol. 2013; 228(8):1665–1675. 10.1002/jcp.24266 23086799

[17] Buresova V, Hajdusek O, Franta Z, Sojka D, Kopacek P. IrAM-An a2-macroglobulin from the hard tick Ixodes ricinus: Characterization and function in phagocytosis of a potential pathogen *Chryseobacterium indologenes*. Dev Comp Immunol. 2009; 33(4):489–498. 10.1016/j.dci.2008.09.011 18948134

[18] Eklund KK, Niemi K, Kovanen PT. Immune functions of serum amyloid A. Crit Rev Immunol. 2012; 32(4):335–348. 10.1615/critrevimmunol.v32.i4.40 . 23237509

[19] Pavan Kumar N, Anuradha R, Andrade BB, Suresh N, Ganesh R, Shankar J, et al Circulating biomarkers of pulmonary and extrapulmonary tuberculosis in children. Clin Vaccine Immunol. 2013; 20(5):704–711. 10.1128/CVI.00038-13 23486418 PMC3647760

[20] Mayer-Barber KD, Andrade BB, Oland SD, Amaral EP, Barber DL, Gonzales J, et al Host-directed therapy of tuberculosis based on interleukin-1 and type I interferon crosstalk. Nat. 2014; 511(7507):99–103. 10.1038/nature13489 24990750 PMC4809146

[21] Gabay C, Kushner I. Acute-phase proteins and other systemic responses to inflammation. N Engl J Med. 1999; 340(6):448–454. 10.1056/NEJM199902113400607 9971870

[22] Jiang TT, Shi LY, Wei LL, Li X, Yang S, Wang C, et al Serum amyloid A, protein Z, and C4b-binding protein β chain as new potential biomarkers for pulmonary tuberculosis. PLoS One. 2017; 12(3):e0173304 10.1371/journal.pone.0173304 28278182 PMC5344400

[23] Gilstad CW. Anaphylactic transfusion reactions. Current Opinion in Hematol. 2003; 10(6):419–423. 10.1097/00062752-200311000-00004 14564171

[24] Grange JM, Kardjito T, Beck JS, Ebeid O, Köhler W, Prokop O. Haptoglobin: an immunoregulatory role in tuberculosis? Tubercle. 1985; 66(1):41–47. 10.1016/0041-3879(85)90052-2 3984037

[25] Schaer DJ, Vinchi F, Ingoglia G, Tolosano E, Buehler PW. Haptoglobin, hemopexin, and related defence pathways-basic science, clinical perspectives, and drug development. Front Physiol. 2014; 5:415 10.3389/fphys.2014.00415 25389409 PMC4211382

[26] De Beer FC, Nel AE, Gie RP, Donald PR, Strachan AF. Serum amyloid A protein and C-reactive protein levels in pulmonary tuberculosis: relationship to amyloidosis. Thorax. 1984; 39(3): 196–200. 10.1136/thx.39.3.196 6710428 PMC459761

[27] Miranda P, Gil-Santana L, Oliveira MG, Mesquita ED, Silva E, Rauwerdink A, et al Sustained elevated levels of C-reactive protein and ferritin in pulmonary tuberculosis patients remaining culture positive upon treatment initiation. PLoS One. 2017; 12(4):e0175278 10.1371/journal.pone.0175278 28384354 PMC5383283

[28] Ruiz-González A, Utrillo L, Bielsa S, Falguera M. Porcel JM. The diagnostic value of serum c-reactive protein for identifying pneumonia in hospitalized patients with acute respiratory symptoms. J Biomarkers. 2016; 2016:2198745 10.1155/2016/2198745 27610265 PMC5004021

[29] Grange JM, Kardjito T, Setiabudi I. A study of acute-phase reactant proteins in Indonesian patients with pulmonary tuberculosis. Tubercle. 1984; 65(1):23–39. 10.1016/0041-3879(84)90027-8 6428016

[30] Walzl G, Ronacher K, Hanekom W, Scriba TJ, Zumla A. Immunological biomarkers of tuberculosis. Nat Rev Immunol. 2011; 11(5):343–354. 10.1038/nri2960 21475309

[31] Adedapo KS, Arinola OG, Ige OM, Adedapo ADA. Combination of reduced levels of serum albumin and alpha-2-macroglobulin differentiates newly diagnosed pulmonary tuberculosis patients from patients on chemotherapy. Afr J Biomed Res. 2006; 9:169–172. 10.4314/ajbr.v9i3.48902

[32] Phalane KG, Kriel M, Loxton AG, Menezes A, Stanley K, van der Spuy GD, et al “Differential expression of host biomarkers in saliva and serum samples from individuals with suspected pulmonary tuberculosis”. Med of Inflammat. 2013; 2013:981984 10.1155/2013/981984 24327799 PMC3845251

[33] Gruys E, Toussaint MJM, Niewold TA, Koopmans SJ. “Acute phase reaction and acute phase proteins”. J Zhejiang Uni Sci B. 2005; 6(11):1045–1056. 10.1631/jzus.2005.B1045 16252337 PMC1390650

[34] Raynes JG, Cooper EH. “Comparison of serum amyloid A protein and C-reactive protein concentrations in cancer and non-malignant disease”. J Clin Pathol. 1983; 36(7):798–803. 10.1136/jcp.36.7.798 6863571 PMC498391

[35] Jai S, Gautam V, Naseem S. Acute-phase proteins: As diagnostic tool. J Pharm Bioall Sci. 2011; 3(1):118–127. 10.4103/0975-7406.76489 21430962 PMC3053509

[36] Wallis RS, Wang C, Doherty TM, Onyebujoh P, Vahedi M, Laang H, et al Biomarkers for tuberculosis disease activity, cure, and relapse. Lancet Infect Dis. 2010; 10(2):68–69. 10.1016/S1473-3099(10)70003-7 20113972

[37] Wilson D, Badri M, Maartens G. Performance of serum C-reactive protein as a screening test for smear-negative tuberculosis in an ambulatory high HIV prevalence population. PLoS One. 2011; 6(1):e15248 10.1371/journal.pone.0015248 21249220 PMC3018418

